# 6,6′-Dimeth­oxy-2,2′-{[(*E*,*E*)-hydrazine-1,2-diyl­idene]bis­(methanylyl­idene)}diphenol methanol disolvate

**DOI:** 10.1107/S1600536812034940

**Published:** 2012-08-15

**Authors:** Nicholas M. Randell, Laurence K. Thompson, Louise N. Dawe

**Affiliations:** a Department of Chemistry, Memorial University of Newfoundland, St Johns, NL, Canada A1B 3X7; b Department of Chemistry and C-CART X-Ray Diffraction Laboratory, Memorial University of Newfoundland, St Johns, NL, Canada A1B 3X7

## Abstract

The title compound, C_16_H_16_N_2_O_4_·2CH_3_OH, is a hydrazone in an *E* geometric arrangement, with an inversion centre at the mid-point of the N—N bond. A symmetry-related pair of six-membered hydrogen-bonded rings [graph-set motif *S* ^1^_1_(6)] are present for the terminal vanillin–imine moieties. Two lattice methanol solvent mol­ecules are present per formula unit (*Z*′ = 1/2), which form hydrogen-bonded chains along [010] with two orientations due to disorder of the methanol H-atom.

## Related literature

The synthesis of the title compound was originally reported by Lin *et al.* (2009 ▶); however, in this study, it was obtained from (2*Z*,6*Z*,*N*′2*E*,*N*′6*E*)-*N*′2,*N*′6-*bis­*(2-hy­droxy-3-meth­oxy­benzyl­idene)pyridine-2,6-*bis­*(carbohydrazonic) acid (Vadavi *et al.* 2011 ▶). The title compound has been used in the synthesis of first-row transition metal complexes (Zou *et al.* 2011 ▶) and in the synthesis of lanthanide complexes (Davidson *et al.* 2006 ▶). A solvent-free structure of the title compound has been previously reported (Lu *et al.* 2011 ▶) and contains a similar intra­molecular hydrogen-bonding motif (Bernstein *et al.* 1995 ▶, Etter *et al.* 1990 ▶) to that reported herein.
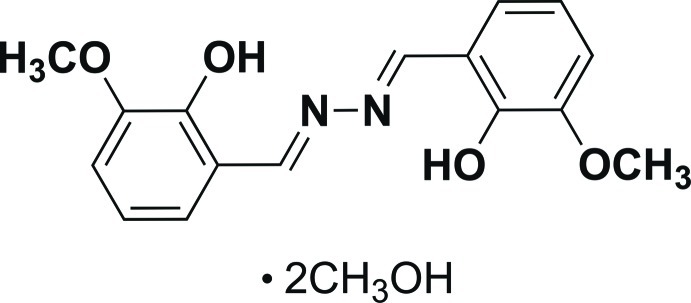


## Experimental

### 

#### Crystal data

C_16_H_16_N_2_O_4_·2CH_4_O
*M* *_r_* = 364.39
Monoclinic, 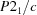

*a* = 20.517 (2) Å
*b* = 4.8374 (5) Å
*c* = 21.366 (3) Å
β = 153.566 (7)°
*V* = 944.0 (3) Å^3^
*Z* = 2
Mo *K*α radiation
μ = 0.10 mm^−1^
*T* = 163 K
0.59 × 0.14 × 0.13 mm

#### Data collection

Rigaku Saturn70 CCD diffractometer
Absorption correction: multi-scan (REQAB; Jacobson, 1998 ▶) *T*_min_ = 0.725, *T*_max_ = 1.000
12068 measured reflections
1941 independent reflections
1651 reflections with *I* > 2σ(*I*)
*R*_int_ = 0.036

#### Refinement

*R*[*F* ^2^ > 2σ(*F* ^2^)] = 0.062
*wR*(*F* ^2^) = 0.194
*S* = 1.09
1941 reflections
129 parameters
4 restraints
H atoms treated by a mixture of independent and constrained refinement
Δρ_max_ = 0.32 e Å^−3^
Δρ_min_ = −0.41 e Å^−3^

### 

Data collection: *CrystalClear-SM Expert* (Rigaku, 2009 ▶); cell refinement: *CrystalClear-SM Expert*; data reduction: *CrystalClear-SM Expert*; program(s) used to solve structure: *SHELXS97* (Sheldrick, 2008 ▶); program(s) used to refine structure: *SHELXL97* (Sheldrick, 2008 ▶); molecular graphics: *OLEX2* (Dolomanov *et al.*, 2009 ▶); software used to prepare material for publication: *OLEX2*.

## Supplementary Material

Crystal structure: contains datablock(s) global, I. DOI: 10.1107/S1600536812034940/hg5235sup1.cif

Structure factors: contains datablock(s) I. DOI: 10.1107/S1600536812034940/hg5235Isup2.hkl

Additional supplementary materials:  crystallographic information; 3D view; checkCIF report

## Figures and Tables

**Table 1:** Hydrogen-bond geometry (Å, °)

*D*—H⋯*A*	*D*—H	H⋯*A*	*D*⋯*A*	*D*—H⋯*A*
O2—H2⋯N1	0.88 (3)	1.77 (7)	2.613 (5)	160 (3)
O3—H3*A*⋯O3^i^	0.89 (8)	1.91 (7)	2.754 (4)	157 (9)
O3—H3*B*⋯O3^ii^	0.88 (7)	1.98 (7)	2.715 (4)	140 (5)

Symmetry codes: (i) 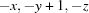; (ii) 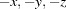.

**Fig. 1.**
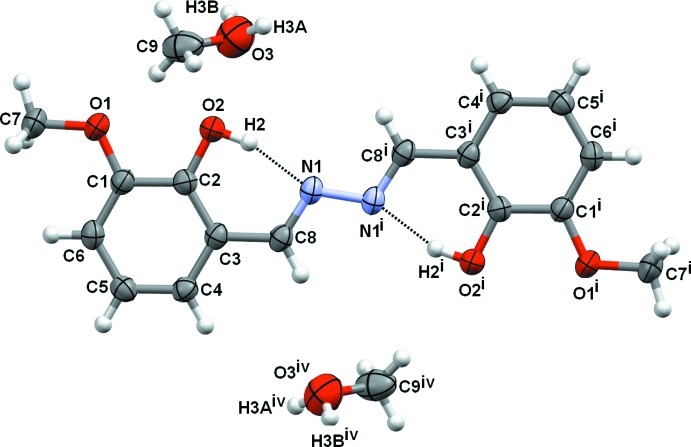
Molecular structure of the title compound at 50% probability ellipsoid level. Symmetry operator i = 1 - x, 2 - y, 1 - z; iv = 1 - x, 1 - y, 1 - z.

**Fig. 2.**
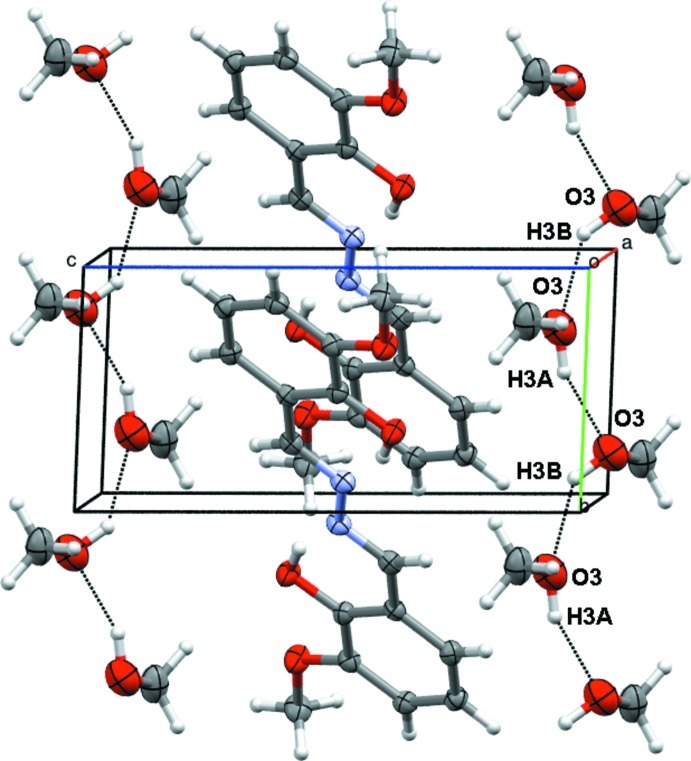
Packed unit cell of the title compound, highlighting the hydrogen bonding chains which propagate along [0 1 0]. One of the disordered H-atoms is omitted from each methanol molecule.

## supplementary crystallographic information

### Comment

The synthesis of this compound was performed because of its intriguing interactions with both transition and lanthanide metals. The ability for the vanillin moieties to bridge multiple metal centers to form polymetallic clusters has potential applications in the fields of molecular magnetics and supramolecular chemistry.

While the previously reported solventless structure (Lu *et al.* 2011) and the title compound are comprised of two vanillin-imine moieties, in the title compound, these are related by an inversion centre about the N1^i^—N1 bond (i = 1 - *x*,2 - *y*,1 - *z*). There are two available geometries for the hydrazone functional group, the *E* and *Z* isomers. Both this report, and the previously reported structure, feature the *E* isomer (Figure 1).

There are multiple hydrogen bonding interactions, one intramolecular for the main molecule and one intermolecular hydrogen bonding chain, however, there are no discernable hydrogen bonding interaction between the main molecule and the solvent methanol molecules. The intramolecular hydrogen bond between N1 and H1 forms a stable six-membered hydrogen bound ring with graph set notation S^1^_1_(6) (one hydrogen bond donor, and one acceptor enclosed in a six-membered ring; Figure 1) (Bernstein *et al.* 1995, Etter *et al.* 1990). This interaction was also present in the previous report (Lu *et al.* 2011). This structural motif would likely provide an energetic barrier to rotation about the C2—C3 bond, which would be required to maximize the number of metals that could be incorporated into a cluster for further study.

A second hydrogen bond interaction is present as a one-dimensional chain of solvent methanol molecules, with graph set notation of C^1^_1_(2) (Figure 2.) The methanolic protons, H3A and H3B, are disordered, with occupancy of 1/2, and when extended packing diagrams are examined, it can be seen that the hydrogen bonded chains propagate along [0 1 0] (*i.e*. run parallel to the *b* axis), with the disorder representing a reversal in the D—H···A orientation.

### Experimental

DyCl_3_.H_2_O (0.06 g; 0.2 mmol) was dissolved in methanol (20 mL). (2*Z*,6*Z*,*N*'2*E*,*N*'6*E*)-*N*'2,*N*'6-*bis*(2-hydroxy-3-methoxybenzylidene)pyridine-2,6-*bis*(carbohydrazonic) acid (Vadavi *et al.* 2011) (0.05 g; 0.1 mmol) was added and the solution was refluxed for 3 h. Upon cooling, the resulting solution was gravity filtered and left for slow evaporation at room temperature to yield yellow-orange crystals of the title compound.

### Refinement

With the exception of H3A, H3B and H2, all hydrogen positions were calculated after each cycle of refinement using a riding model, with C—H = 0.93 Å and *U*_iso_(H) = 1.2*U*_eq_(C) for aromatic H atoms, and with C—H = 0.96 Å and *U*_iso_(H) = 1.5*U*_eq_(C) for methyl H atoms. H2 was allowed to refine positionally, with *U*_iso_(H) = 1.5*U*_eq_(O2). The disordered H atoms, H3A and H3B were refined with distance (O3—H) and angle (C5—O3—H) restraints of 0.87 Å and 110° respectively, and with *U*_iso_(H) = 1.5*U*_eq_(O3). H3A and H3B were both assigned an occupancy of 0.5 based on similar residual peak height from examination of difference maps, prior to their introduction.

### Crystal data

**Table:**

C_16_H_16_N_2_O_4_·2CH_4_O	*F*(000) = 388
*M**_r_* = 364.39	*D*_x_ = 1.282 Mg m^−^^3^
Monoclinic, *P*2_1_/*c*	Mo *K*α radiation, λ = 0.71075 Å
Hall symbol: -P 2ybc	Cell parameters from 9154 reflections
*a* = 20.517 (2) Å	θ = 3.4–27.6°
*b* = 4.8374 (5) Å	µ = 0.10 mm^−^^1^
*c* = 21.366 (3) Å	*T* = 163 K
β = 153.566 (7)°	Irregular, yellow
*V* = 944.0 (3) Å^3^	0.59 × 0.14 × 0.13 mm
*Z* = 2	

### Data collection

**Table:**

Rigaku Saturn70 CCD diffractometer	1941 independent reflections
Radiation source: fine-focus sealed tube	1651 reflections with *I* > 2σ(*I*)
Graphite - Rigaku SHINE monochromator	*R*_int_ = 0.036
Detector resolution: 14.63 pixels mm^-1^	θ_max_ = 26.5°, θ_min_ = 3.4°
ω scans	*h* = −25→25
Absorption correction: multi-scan (REQAB; Jacobson, 1998)	*k* = −5→6
*T*_min_ = 0.725, *T*_max_ = 1.000	*l* = −26→26
12068 measured reflections	

### Refinement

**Table:**

Refinement on *F*^2^	Primary atom site location: structure-invariant direct methods
Least-squares matrix: full	Secondary atom site location: difference Fourier map
*R*[*F*^2^ > 2σ(*F*^2^)] = 0.062	Hydrogen site location: inferred from neighbouring sites
*wR*(*F*^2^) = 0.194	H atoms treated by a mixture of independent and constrained refinement
*S* = 1.09	*w* = 1/[σ^2^(*F*_o_^2^) + (0.118*P*)^2^ + 0.3328*P*] where *P* = (*F*_o_^2^ + 2*F*_c_^2^)/3
1941 reflections	(Δ/σ)_max_ < 0.001
129 parameters	Δρ_max_ = 0.32 e Å^−^^3^
4 restraints	Δρ_min_ = −0.41 e Å^−^^3^

### Special details

**Table:**

Geometry. All e.s.d.'s (except the e.s.d. in the dihedral angle between two l.s. planes) are estimated using the full covariance matrix. The cell e.s.d.'s are taken into account individually in the estimation of e.s.d.'s in distances, angles and torsion angles; correlations between e.s.d.'s in cell parameters are only used when they are defined by crystal symmetry. An approximate (isotropic) treatment of cell e.s.d.'s is used for estimating e.s.d.'s involving l.s. planes.
Refinement. Refinement of *F*^2^ against ALL reflections. The weighted *R*-factor *wR* and goodness of fit *S* are based on *F*^2^, conventional *R*-factors *R* are based on *F*, with *F* set to zero for negative *F*^2^. The threshold expression of *F*^2^ > σ(*F*^2^) is used only for calculating *R*-factors(gt) *etc*. and is not relevant to the choice of reflections for refinement. *R*-factors based on *F*^2^ are statistically about twice as large as those based on *F*, and *R*- factors based on ALL data will be even larger.

### Fractional atomic coordinates and isotropic or equivalent isotropic displacement parameters (Å^2^)

**Table:**

	*x*	*y*	*z*	*U*_iso_*/*U*_eq_	Occ. (<1)
O2	0.26395 (15)	0.6929 (3)	0.38898 (14)	0.0446 (4)	
H2	0.318 (3)	0.797 (6)	0.411 (3)	0.067*	
C3	0.51958 (19)	0.5637 (4)	0.61567 (19)	0.0321 (4)	
C4	0.6264 (2)	0.3959 (4)	0.73588 (19)	0.0360 (5)	
H4	0.7240	0.4131	0.8082	0.043*	
C2	0.37270 (19)	0.5380 (4)	0.50753 (18)	0.0341 (5)	
C1	0.3337 (2)	0.3422 (4)	0.52005 (19)	0.0357 (5)	
C6	0.4408 (2)	0.1796 (4)	0.6393 (2)	0.0367 (5)	
H6	0.4151	0.0514	0.6478	0.044*	
C5	0.5869 (2)	0.2059 (4)	0.74696 (19)	0.0381 (5)	
H5	0.6580	0.0949	0.8265	0.046*	
O1	0.18781 (15)	0.3354 (3)	0.40867 (15)	0.0490 (5)	
N1	0.46904 (17)	0.9117 (3)	0.49594 (16)	0.0353 (4)	
C8	0.5634 (2)	0.7607 (4)	0.60529 (19)	0.0347 (4)	
H8	0.6619	0.7772	0.6796	0.042*	
C7	0.1407 (2)	0.1307 (5)	0.4117 (2)	0.0498 (6)	
H7A	0.0360	0.1364	0.3253	0.075*	
H7B	0.1839	0.1667	0.4890	0.075*	
H7C	0.1704	−0.0487	0.4229	0.075*	
O3	0.0380 (3)	0.2477 (5)	0.0577 (3)	0.0813 (7)	
H3A	0.032 (10)	0.428 (5)	0.044 (9)	0.122*	0.50
H3B	−0.024 (7)	0.138 (13)	−0.015 (5)	0.122*	0.50
C9	0.0339 (3)	0.2220 (7)	0.1198 (3)	0.0704 (8)	
H9A	−0.0661	0.2228	0.0478	0.106*	
H9B	0.0853	0.3742	0.1827	0.106*	
H9C	0.0796	0.0516	0.1713	0.106*	

### Atomic displacement parameters (Å^2^)

**Table:**

	*U*^11^	*U*^22^	*U*^33^	*U*^12^	*U*^13^	*U*^23^
O2	0.0338 (7)	0.0509 (9)	0.0391 (8)	0.0011 (6)	0.0315 (7)	0.0087 (6)
C3	0.0351 (9)	0.0337 (10)	0.0352 (9)	−0.0036 (7)	0.0323 (8)	−0.0044 (7)
C4	0.0330 (9)	0.0410 (10)	0.0353 (9)	−0.0003 (7)	0.0308 (9)	−0.0011 (8)
C2	0.0345 (9)	0.0356 (10)	0.0340 (9)	0.0001 (7)	0.0309 (9)	0.0006 (7)
C1	0.0339 (9)	0.0396 (10)	0.0364 (9)	−0.0047 (8)	0.0318 (9)	−0.0036 (8)
C6	0.0442 (10)	0.0364 (10)	0.0427 (10)	−0.0035 (8)	0.0404 (10)	−0.0019 (8)
C5	0.0406 (10)	0.0400 (10)	0.0367 (9)	0.0033 (8)	0.0349 (9)	0.0035 (8)
O1	0.0352 (7)	0.0593 (10)	0.0456 (8)	−0.0025 (6)	0.0354 (7)	0.0066 (7)
N1	0.0399 (8)	0.0368 (9)	0.0410 (9)	−0.0055 (7)	0.0376 (8)	−0.0037 (7)
C8	0.0370 (9)	0.0376 (10)	0.0389 (9)	−0.0043 (7)	0.0351 (9)	−0.0050 (8)
C7	0.0427 (11)	0.0598 (14)	0.0506 (12)	−0.0086 (10)	0.0422 (11)	−0.0013 (10)
O3	0.0841 (14)	0.0932 (16)	0.0861 (14)	−0.0099 (12)	0.0783 (14)	−0.0147 (13)
C9	0.0518 (13)	0.098 (2)	0.0550 (14)	0.0091 (14)	0.0472 (13)	0.0002 (14)

### Geometric parameters (Å, º)

**Table:**

O2—C2	1.354 (2)	O1—C7	1.425 (3)
O2—H2	0.88 (3)	N1—C8	1.281 (3)
C3—C2	1.402 (3)	N1—N1^i^	1.407 (3)
C3—C4	1.407 (3)	C8—H8	0.9300
C3—C8	1.456 (3)	C7—H7A	0.9600
C4—C5	1.378 (3)	C7—H7B	0.9600
C4—H4	0.9300	C7—H7C	0.9600
C2—C1	1.412 (3)	O3—C9	1.409 (4)
C1—O1	1.367 (2)	O3—H3A	0.89 (2)
C1—C6	1.383 (3)	O3—H3B	0.88 (2)
C6—C5	1.395 (3)	C9—H9A	0.9600
C6—H6	0.9300	C9—H9B	0.9600
C5—H5	0.9300	C9—H9C	0.9600
			
C2—O2—H2	98.1 (19)	C8—N1—N1^i^	113.50 (19)
C2—C3—C4	119.54 (17)	N1—C8—C3	121.36 (17)
C2—C3—C8	121.05 (17)	N1—C8—H8	119.3
C4—C3—C8	119.41 (16)	C3—C8—H8	119.3
C5—C4—C3	120.29 (17)	O1—C7—H7A	109.5
C5—C4—H4	119.9	O1—C7—H7B	109.5
C3—C4—H4	119.9	H7A—C7—H7B	109.5
O2—C2—C3	122.92 (17)	O1—C7—H7C	109.5
O2—C2—C1	117.48 (16)	H7A—C7—H7C	109.5
C3—C2—C1	119.59 (17)	H7B—C7—H7C	109.5
O1—C1—C6	125.69 (17)	C9—O3—H3A	106 (3)
O1—C1—C2	114.56 (16)	C9—O3—H3B	113 (4)
C6—C1—C2	119.74 (17)	H3A—O3—H3B	118 (7)
C1—C6—C5	120.61 (17)	O3—C9—H9A	109.5
C1—C6—H6	119.7	O3—C9—H9B	109.5
C5—C6—H6	119.7	H9A—C9—H9B	109.5
C4—C5—C6	120.23 (17)	O3—C9—H9C	109.5
C4—C5—H5	119.9	H9A—C9—H9C	109.5
C6—C5—H5	119.9	H9B—C9—H9C	109.5
C1—O1—C7	117.07 (16)		

Symmetry code: (i) −*x*+1, −*y*+2, −*z*+1.

### Hydrogen-bond geometry (Å, º)

**Table:**

*D*—H···*A*	*D*—H	H···*A*	*D*···*A*	*D*—H···*A*
O2—H2···N1	0.88 (3)	1.77 (7)	2.613 (5)	160 (3)
O3—H3*A*···O3^ii^	0.89 (8)	1.91 (7)	2.754 (4)	157 (9)
O3—H3*B*···O3^iii^	0.88 (7)	1.98 (7)	2.715 (4)	140 (5)

Symmetry codes: (ii) −*x*, −*y*+1, −*z*; (iii) −*x*, −*y*, −*z*.
